# Identification of Intragenic Exon Deletions and Duplication of *TCF12* by Whole Genome or Targeted Sequencing as a Cause of *TCF12*‐Related Craniosynostosis

**DOI:** 10.1002/humu.23010

**Published:** 2016-06-02

**Authors:** Jacqueline A.C. Goos, Aimee L. Fenwick, Sigrid M.A. Swagemakers, Simon J. McGowan, Samantha J.L. Knight, Stephen R.F. Twigg, A. Jeannette M. Hoogeboom, Marieke F. van Dooren, Frank J. Magielsen, Steven A. Wall, Irene M.J. Mathijssen, Andrew O.M. Wilkie, Peter J. van der Spek, Ans M.W. van den Ouweland

**Affiliations:** ^1^ Erasmus MC, Department of Plastic and Reconstructive Surgery and Hand SurgeryUniversity Medical Center RotterdamRotterdamThe Netherlands; ^2^ Clinical Genetics Group, Weatherall Institute of Molecular MedicineUniversity of OxfordJohn Radcliffe HospitalOxfordUK; ^3^ Erasmus MC, Department of BioinformaticsUniversity Medical Center RotterdamRotterdamThe Netherlands; ^4^ Computational Biology Research Group, Weatherall Institute of Molecular MedicineUniversity of OxfordJohn Radcliffe HospitalOxfordUK; ^5^ NIHR Biomedical Research Centre, Wellcome Trust Centre for Human GeneticsUniversity of OxfordOxfordUK; ^6^ Erasmus MC, Department of Clinical GeneticsUniversity Medical Center RotterdamRotterdamThe Netherlands; ^7^ Craniofacial Unit, Department of Plastic and Reconstructive SurgeryOxford University Hospitals NHS TrustJohn Radcliffe HospitalOxfordUK

**Keywords:** *TCF12*‐related craniosynostosis, intragenic exon deletion, exon duplication, rearrangements

## Abstract

*TCF12*‐related craniosynostosis can be caused by small heterozygous loss‐of‐function mutations in *TCF12*. Large intragenic rearrangements, however, have not been described yet. Here, we present the identification of four large rearrangements in *TCF12* causing *TCF12*‐related craniosynostosis. Whole‐genome sequencing was applied on the DNA of 18 index cases with coronal synostosis and their family members (43 samples in total). The data were analyzed using an autosomal‐dominant disease model. Structural variant analysis reported intragenic exon deletions (of sizes 84.9, 8.6, and 5.4 kb) in *TCF12* in three different families. The results were confirmed by deletion‐specific PCR and dideoxy‐sequence analysis. Separately, targeted sequencing of the *TCF12* genomic region in a patient with coronal synostosis identified a tandem duplication of 11.3 kb. The pathogenic effect of this duplication was confirmed by cDNA analysis. These findings indicate the importance of screening for larger rearrangements in patients suspected to have *TCF12*‐related craniosynostosis.

Craniosynostosis is a condition in which the calvarial sutures are fused prematurely. Fusion of the coronal sutures has the highest chance of having a specific genetic cause. In patients with coronal synostosis, mutations are often found in *FGFR2* (MIM #176943), *FGFR3* (MIM #134934), *TWIST1* (MIM #601622), and *EFNB1* (MIM #300035). Recently, however, another disease gene for coronal synostosis, *TCF12* (MIM #600480), has been identified [Sharma et al., 2013]. The product of this gene is a member of the basic helix‐loop‐helix E‐protein family and forms heterodimers with TWIST1 [Connerney et al., 2006; Sharma et al., 2013].

In 32% and 10%, respectively, of patients with bicoronal and unicoronal synostosis in whom other genetic testing was negative, a pathogenic heterozygous mutation could be identified in *TCF12* by dideoxy‐sequencing [Sharma et al., 2013]. In addition to coronal synostosis, patients can have craniofacial features suggestive of Saethre–Chotzen syndrome, and a minority have developmental delay and/or learning disabilities. On the other hand, a substantial proportion (>50%) of individuals heterozygous for a pathogenic *TCF12* mutations are nonpenetrant [Sharma et al., 2013].

In patients with *TCF12*‐related craniosynostosis, point mutations are predominantly found [Sharma et al., 2013; di Rocco et al., 2014; Paumard‐Hernandez et al., 2014]. A few patients with craniosynostosis and intellectual disability have been reported with large chromosome 15q deletions including *TCF12* [Fukushima et al., 1990; Shur et al., 2003; Hiraki et al., 2008]. Recently, Le Tanno et al. (2014) have described a heterozygous de novo deletion of 3.64 Mb due to an unbalanced maternally inherited translocation in a patient with coronal craniosynostosis and intellectual disability. In addition, a 72‐year‐old patient has been identified with intellectual disability (without clear signs of craniosynostosis) and a *TCF12* microdeletion of 84–121 kb, removing exons 19–21 and extending 3′ from the end of the gene [Piard et al., 2015]. Gross rearrangements with both breakpoints lying within *TCF12*, however, have not been described to date; in the original report of *TCF12* mutations, an assay for deletions using multiplex‐ligation‐dependent probe amplification (MLPA) found no rearrangements in 226 mixed craniosynostosis samples negative for intragenic *TCF12* mutations [Sharma et al., 2013].

In this study, we describe the identification of three large inherited intragenic exon deletions in *TCF12* using whole‐genome sequencing (WGS) and one large inherited duplication using targeted *TCF12* sequencing.

Within the framework of a broader study into the genetic causes of craniosynostosis, WGS was applied to DNA of 18 Dutch index cases with coronal synostosis and negative testing for *FGFR2*, *FGFR3*, and *TWIST1*, and their family members (43 samples in total), by Complete Genomics, a BGI company (Mountain View, CA), as described in Drmanac et al. (2010). The data were analyzed using an autosomal‐dominant disease model. Structural variant analysis using a custom made Python script [Gilissen et al., 2014] reported different large intragenic exon deletions of *TCF12* in three families (families 1, 2, and 3). The clinical features of the families are summarized in Table 1 and pedigrees and full subject descriptions are provided in the Supp. Materials and Methods (Supp. Fig. S1 and subject descriptions, respectively).

**Table 1:** Clinical Features of Families Harboring *TCF12* Rearrangements

	Family 1	Family 2	Family 3	Family 4
cDNA position of rearrangement	c.391–5871_1746–1795del	c.1745 + 4562_1978 + 3153del	c.1979–3147_*12–1497del	c.1746–1697_*11 + 65dup
Size of rearrangement (kb)	84.9	8.6	5.4	11.3
Exons involved	7–18	19	20	19 and 20
Type of rearrangement	Deletion	Deletion	Deletion	Duplication
Gender of index patient	Male	Female	Male	Male
Gestational age (weeks)	37+6	42+4	39+4	38
Birth weight (grams)	3,075	3,620	3,365	NA
Cranial suture fusion	RC	BC	All except M	BC
Major craniofacial procedures	Fronto‐supraorbital remodeling @ 10 months	Supraorbital advancement @ 9 months	Fronto‐biparietal remodeling @ 8 months, parieto‐occipital decompression @ 2 years	FOAR @ 10.5 months
Development	Mild learning problems VIQ 82, PIQ 64, GIQ 74	Normal	Normal	Normal
Limbs	Fifth finger camptodactyly	Normal	Normal	Fifth finger clinodactyly
Other major clinical features	Divergent strabismus, recurrent infections, febrile seizures	Myopia, crowding of teeth, divergent growth pattern	Increased ICP	Class II.1 malocclusion, small ears with prominent helical crura
Family history	Mother LC	Sister of father brachycephaly	Negative	Flattened foreheads in maternal half‐brother and his daughter
Previous genetic diagnostics	Karyotyping, *FGFR2*, *FGFR3*, *TWIST1, TCF12*	*FGFR2*, *FGFR3*, *TWIST1, TCF12*	*FGFR2*, *FGFR3, TWIST1*	*FGFR2, FGFR3, TWIST1, TCF12*
Subjects sequenced	II.1, II.2, and III.1	II.1, II.2, II.3, and III.1	II.1, II.2, and III.2	III.1

BC, bicoronal; FOAR, fronto‐orbital advancement and remodeling; GIQ, Global IQ; LC, left coronal; M, metopic; PIQ, performal IQ; RC, right coronal; VIQ, verbal IQ.

A heterozygous 84,949 bp deletion was reported in family 1, starting at chr15:57478485 and ending at chr15:57563433 (c.391‐5871_1746‐1795del) in DNA of the index patient and his mother (using reference NM_207037.1 on GRCh37). Exons 7–18 were deleted. In family 2, a heterozygous deletion of 8,580 bp was reported, starting at chr15:57560034 and ending at chr15:57568613 (c.1745+4562_1978+3153del) in DNA of the index patient, the clinically unaffected father and affected paternal aunt. The deletion removed exon 19. In family 3, a heterozygous 5,363 bp deletion was reported, starting at chr15:57571496 and ending at chr15:57576858 (c.1979‐3147_*12‐1497del) in DNA of the index patient and his clinically unaffected mother. This deleted exon 20. The three deletions were not seen in control samples (i.e., Structural Variation Baseline Genome Set comprising 52 baseline genomes used by Complete Genomics and 588 Wellderly samples [Scripps Wellderly Genome Resource, The Scripps Wellderly Study, La Jolla, CA [December, 2015]], funding provided by Scripps Health and NIH/NCATS UL1 TR00114).

Deletion‐specific PCRs were designed to confirm the findings by WGS (Fig. 1 A–E). In family 1, the mutant PCR product of 644 bp was present in II.2 and III.1 (Fig. 1B, lanes 2–4, and C). An analysis of the normal sequence showed that the proximal breakpoint resided in an *AluJb* element, whereas the distal breakpoint was located in a *L1P2* repetitive element. The sequences of the breakpoints showed no significant similarity except for a shared 4 bp GAGC motif. In family 2, the mutant PCR product of 556 bp was present in II.1, II.2, and III.1 (Fig. 1B, lanes 5–8, and D). The proximal breakpoint was located in an *MIRb* element and the distal breakpoint in a *HAL1* element. The sequences showed no significant similarity, except for a shared cytosine. In family 3, the mutant PCR product of 646 bp was present in II.2 and III.2 (Fig. 1B, lanes 9–11, and E). The proximal breakpoint also resided in a *HAL1* element; the distal breakpoint was not located in a repetitive element. However, an *AluSz* repeat was located 151 bp centromeric of the distal breakpoint and an *MIRb* 789 bp telomeric. The sequences of the breakpoints showed no significant similarity. Amplification of patient cDNA of family 3 with primers located in exon 18 and 3′ UTR (exon 21) showed an extra smaller product, absent in control cDNA. Sequencing of the smaller‐sized product indicated skipping of exon 20 (Supp. Fig. S2).

**Figure 1:**
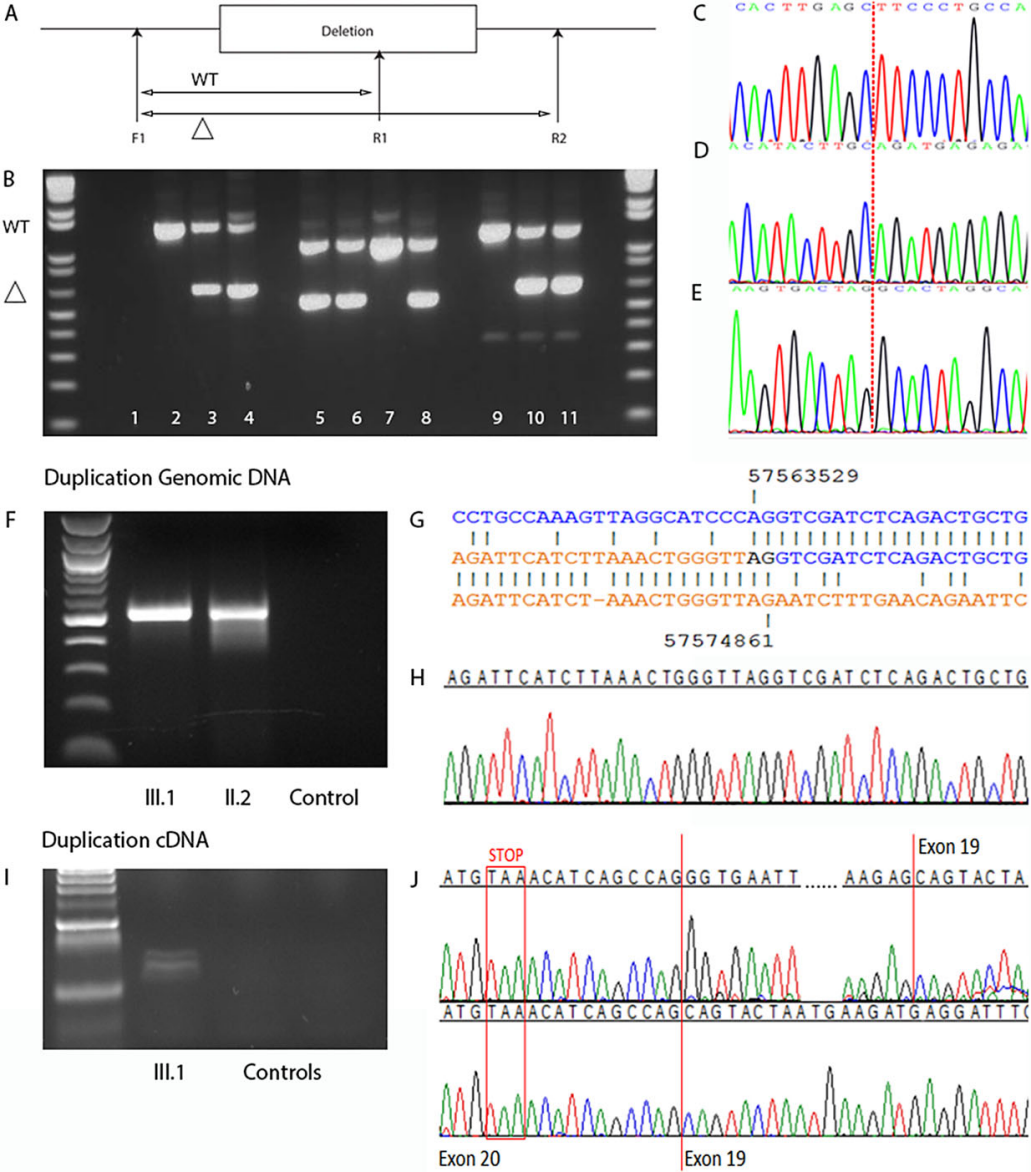
Rearrangements identified in TCF12. **A**–**E**: Confirmation of deletions. A primer design. F1, forward primer; R1, reverse primer 1; R2, reverse primer 2. F1+R1, wild‐type allele (WT); F1+R2, mutant allele (Δ). **B**: Results of mutation‐specific deletion PCR analysis. Lane 1, negative control. Lanes 2–4, family 1; wild‐type fragment 1,455 bp, mutant fragment 644 bp; lane 2, II.1; lane 3, II.2; lane 4, III.1. Lanes 5–8, family 2; wild‐type fragment 1,107 bp, mutant fragment 556 bp; lane 5, II.1; lane 6, II.2; lane 7, II.3; lane 8, III.1. Lanes 9–11, family 3; wild‐type fragment 1,303 bp, mutant fragment 646 bp; lane 9, II.1; lane 10, II.2; lane 11, III.2. **C–E**: Electropherograms of dideoxy sequence analysis. **C**: Mutant allele in family 1, exons 7–18 deleted. **D**: Mutant allele in family 2, exon 19 deleted. **E**: Mutant allele in family 3, exon 20 deleted. Dashed lines indicate where deletions occurred. **F–J**: Confirmation of duplication. **F**: PCR from genomic DNA confirming the presence of duplication in index patient of family 4 (III.1), which was inherited from the index patient's clinically unaffected mother (II.2). **G**: The sequence at the duplication breakpoint is sandwiched between the normal proximal (above) and distal (below) sequences, with the electropherogram underneath (**H**). **I**: cDNA amplified from the index patient (III.1) with primers specific to the mutant allele. Two different products were visible on the gel (but absent in two control cDNA samples). **J**: Sequencing of these products indicated splicing from exon 20 to the duplicated exon 19 in the smaller‐sized product (lower electropherogram). The larger product (upper electropherogram) contains an additional 35 nucleotide neo‐exon between exons 20 and 19. The normal stop codon in exon 20 is highlighted.

In parallel to this work, targeted sequencing of DNA samples of 160 British unrelated subjects with craniosynostosis, including the coronal suture, and previously negative testing of the *FGFR2*, *FGFR3*, *TWIST1*, and *TCF12* genes, was carried out by capturing the *TCF12* genomic region (chr15: 57,029,979‐57,670,037) using the SeqCap EZ Choice Library system (Roche‐Nimblegen, Inc., Madison, WI). Using analysis with Pindel [Ye et al., 2009], a heterozygous tandem duplication was called in the index patient of family 4 (pedigree in Supp. Fig. S1D (II.1)).

Breakpoint spanning PCR (using primers orientated in opposite directions in the wild‐type sequence, shown in Supp. Table S1) showed a mutant PCR product from genomic DNA of both the index patient and his clinically unaffected mother (Fig. 1F). Dideoxy sequencing confirmed a tandem duplication of 11,331 base pairs, starting at chr15:57563531 and ending at chr15:57574861 (c.1746‐1697_*11+65dup) in the DNA of the index patient of family 4. The duplication included *TCF12* exons 19 and 20.

An analysis of the normal sequence showed that the proximal breakpoint was located in an *L1P2* repetitive element, whereas the distal breakpoint did not reside in a repetitive element. Further, sequence analysis revealed the presence of an *MER1A* DNA sequence 678 bp centromeric of the distal breakpoint, and the presence of an *AluSx* element and a simple tandem repeat at 597 and 1,165 bp, respectively, telomeric of the distal breakpoint. The sequences of the breakpoints shared an AG dinucleotide (Fig. 1G).

Amplification of patient cDNA with primers located in exon 18 and 3′ UTR (therefore spanning the duplication) showed only a single product corresponding to the wild‐type allele (data not shown). This suggested that the product from the duplicated allele might be expressed at a relatively low level, although bias in PCR amplification favoring the smaller wild‐type product could not be excluded. We therefore designed a pair of primers in exon 19, orientated in opposite directions, predicted to generate a product only from the duplicated allele. Electrophoresis of PCR products revealed two relatively weak fragments in the patient cell line, absent in two controls (Fig. 1I). Sequencing of the smaller‐sized product indicated splicing from exon 20 to the duplicated exon 19, whereas the larger product contained additional neo‐exon sequence of 35 bp, originating from within the intron 18–19 sequence (Fig. 1J).

In summary, we have identified the first intragenic duplication and deletions within *TCF12* in patients with craniosynostosis. These findings demonstrate the importance of screening, not only for point mutations and small indels in *TCF12*, but also for larger rearrangements.

In patients with nonsyndromic and syndromic bicoronal or unicoronal synostosis, *FGFR2*, *FGFR3*, and *TWIST1* are often tested routinely for mutations. In approximately 28% of the craniosynostosis patients (with any combination of sutures fused) having a genetic cause, a pathogenic mutation can be identified in *FGFR2*, in 19% of the patients in *FGFR3*, and in 16% in *TWIST1*. *TCF12* intragenic mutations cause approximately 4% of these cases [Sharma et al., 2013].

To date, we have tested, by dideoxy‐sequencing of *TCF12*, 105 Dutch syndromic and nonsyndromic coronal craniosynostosis index patients with negative results for *FGFR2, FGFR3*, and *TWIST1* testing. This led to the identification of 22 *TCF12* mutation‐positive index cases (including 14 cases that were previously described by Sharma et al. (2013). We now report the identification of gross deletions of *TCF12* in three out of 18 index cases that were analyzed by WGS (in five cases a mutation was found in another gene). Furthermore, we describe a gross *TCF12* duplication in a further patient with coronal synostosis, using targeted sequencing of the *TCF12* genomic region.

The identification of *TCF12* deletions in two of the Dutch patients (families 1 and 2) was a particular surprise, because these patients had been included in the negative MLPA analysis (zero positive from 226 samples) previously reported by Sharma et al. (2013). Re‐examination of the raw MLPA data revealed that the analysis had not been performed correctly, and both deletions were in fact evident. Further scrutiny of the remaining MLPA data revealed an Oxford sample that harbored a deletion of exons 5–19, which was independently found in the *TCF12* capture sequencing data (not shown). Hence, it is evident that *TCF12* deletions contribute more frequently to coronal synostosis than previously thought; combining the results of WGS (3/13) and the reanalyzed MLPA dataset (1/95), four of 108 (3.7%) individuals with this diagnosis and previously negative for standard genetic testing, were found to have deletions. Overall, three out of 25 Dutch cases of *TCF12*‐related craniosynostosis were caused by large, intragenic *TCF12* rearrangements.

Analyzing the breakpoint sequences, it appears that the rearrangements occur in, or in the proximity of, repeat sequences (family 1 *AluJb* SINE and *L1P2* LINE element; family 2 *MIRb* SINE and *HAL1* LINE; family 3 *HAL1* LINE and in proximity of *AluSz* SINE/*MIRb* SINE; family 4 *L1P2* LINE and in proximity of *MER1A*/*AluSx* SINE). Furthermore, the sequences of the breakpoints show no significant similarity (family 1 four‐nucleotide homology; family 2 one shared nucleotide; family 3 none; family 4 two shared nucleotides). The nonobligatory presence of terminal microhomology and repeat sequences indicates classical nonhomologous end joining as the most likely mechanism [Stankiewicz et al., 2003; Gu et al., 2008; Chen et al., 2010].

All but one previously described pathogenic mutations are located in the 3′ half of *TCF12* (exons 9–19) [Sharma et al., 2013]. The first two deletions also include this region of the gene (family 1 exons 7–18 and family 2 exon 19). The deletion in family 1 contains regions of *TCF12* encoding the activation domain 2 and the rep domain. In family 2, the region encoding the functionally important basic helix‐loop‐helix domain is included [Zhang et al., 1991; Aronheim et al., 1993; Markus et al., 2002]. These domains are still present in the mutant alleles in families 3 and 4. cDNA studies indicate skipping of exon 20 in family 3 (Supp. Fig. S2). Although the duplicated allele in family 4 should retain the capacity to translate the full‐length protein, the cDNA studies (Fig. 1G) indicate that this transcript is likely unstable, causing functional haploinsufficiency. The rearrangements in families 3 and 4 are the first mutations described that lie 3′ of the region that encodes the bHLH domain.

While the deletion in family 1 is 10‐fold larger than that in family 2 and removes two functional domains of *TCF12*, the clinical craniofacial features of the index patient of family 1 are not more severe than the features of the index patient of family 2. The index patient of family 1 has unicoronal synostosis, whereas the index patient of family 2 and her aunt have bicoronal synostosis. The index patient of family 1, however, does have mild learning disability. The most severe phenotype in our study is seen in the index patient of family 3 (synostosis of almost all sutures and increased intracranial pressure), though the smallest deletion is present in this family. Although the index patient of family 4 has a duplication partially overlapping the deleted regions of both family 2 and the more severely affected family 3, he only shows bicoronal synostosis and minor anomalies like fifth finger clinodactyly. Clinodactyly and brachydactyly (present in his mother) have previously been described in association with *TCF12* mutations [Sharma et al., 2013; di Rocco et al., 2014]. Overall, the findings do not reveal any genotype–phenotype correlation.

The index patient of family 3 does not exhibit the classical clinical phenotype of *TCF12*‐related craniosynostosis, since all vault sutures were fused except for the metopic suture. Only two patients have been described previously with synostosis of the coronal sutures combined with synostosis of the sagittal suture [Sharma et al., 2013]. Also, the patient had papilledema postoperatively, indicating increased intracranial pressure. To our knowledge, this is the first patient described with *TCF12*‐related craniosynostosis and increased intracranial pressure postoperatively.

In conclusion, the clinical features of the index patients of family 1, 2, and 4 fit the phenotype of *TCF12*‐related craniosynostosis as described by Sharma et al. (2013). The reduced penetrance described previously is seen in three of our families as well. Our study demonstrates that *TCF12*‐related craniosynostosis can also be caused by large intragenic rearrangements and that there is no indication of a genotype–phenotype correlation. Therefore, mutation analysis of *TCF12* should also include a search for larger rearrangements.

*Disclosure statement*: The authors declare no conflict of interest.

## Supporting information

Disclaimer: Supplementary materials have been peer‐reviewed but not copyedited.

Supporting Material
