# Supplementary material for: Identification of Intragenic Exon Deletions and Duplication of TCF12 by Whole Genome or Targeted Sequencing as a Cause of TCF12‐Related Craniosynostosis
Source: Hum Mutat. 2016 Jun 2;37(8):732–6. doi: 10.1002/humu.23010 (PMC4949653; doi:10.1002/humu.23010)
Supplement: Supplementary file 1 — Supporting Material [file HUMU-37-732-s001.pdf]

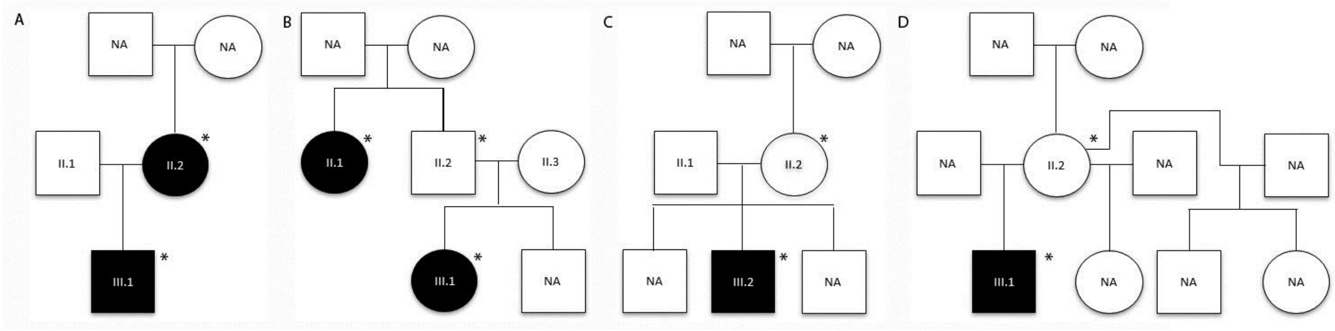

Supp. Figure S1. Pedigrees of family 1 (**A**), family 2 (**B**) family 3 (**C**) and family 4 (**D**). Black is clinically affected, white is unaffected. Asterisks indicate carriers of rearrangements. NA= not available.

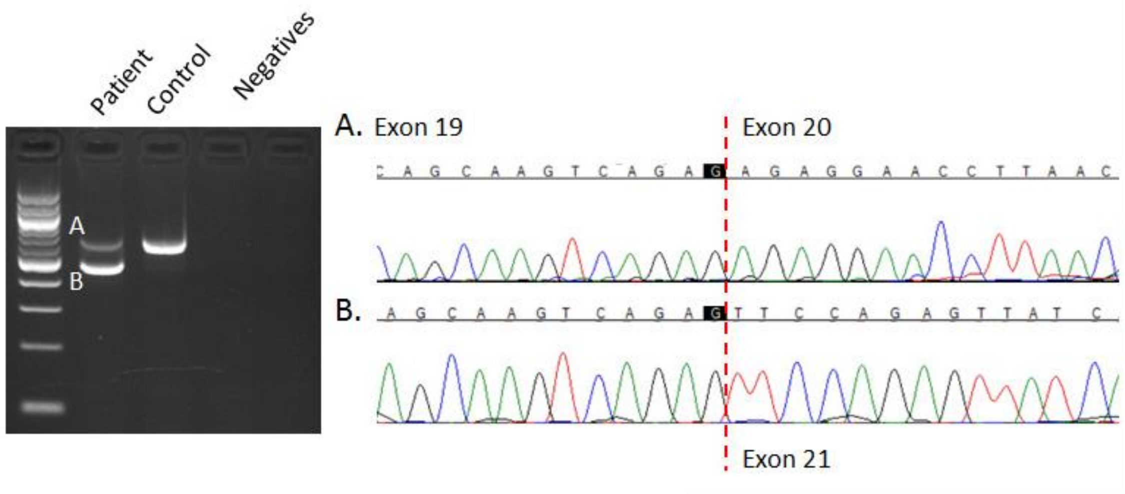

Supp. Figure S2. Amplification of cDNA using primers in *TCF12* exons 18 and 3′ UTR; cDNA amplified from the index patient in family 3 resulted in an extra, smaller product. Sequence traces of gel-purified products from A and B are shown; product B demonstrated skipping of exon 20.

Supp. Table S1. Primers used for deletion PCR, breakpoint spanning PCR and dideoxy-sequencing

Amplicon	5'→ 3'		Product size (bp)
	Forward	Reverse	
Deletion family 1 F1/R1 (WT)	tgtcaaaaagcaaacaaaagtaaac	ggttttcacactgttaccaacaa	1455
Deletion family 1 F1/R2 (M)	tgtcaaaaagcaaacaaaagtaaac	gtatactcctgcccaaatactgc	644
Deletion family 2 F1/R1 (WT)	caaaagtagccaaagtcaaatgg	aaacatatgagggagatgctctg	1107
Deletion family 2 F1/R2 (M)	caaaagtagccaaagtcaaatgg	ttgaaggtgctgctccacta	556
Deletion family 3 F1/R1 (WT)	gggactacaggtgggtaccag	aagagttctaattttctaagcccctgt	1303
Deletion family 3 F1/R2 (M)	gggactacaggtgggtaccag	gctgccctgtctcttgctac	646
Deletion family 3 cDNA	cttgcaaagtcagtctggaactgt	ctgtcaggtttgtgtcttcagataatctg	636 (wild-type)<br>482 (minus exon 20)
Duplication genomic	atcctgggcttagtgaaactaccaaccctatg	cattgccgaggtgggttgcttccaagat	530
Duplication cDNA	cttgcaaagtcagtctggaactgt	ctgtcaggtttgtgtcttcagataatctg	636 (wild-type)
Duplication cDNA mutant specific	cagtcatccttagtctagaacagcaagtc	ctatcttctgttcagggttcaaatcctcatc	231 (predicted)

Primer sequence from 5' to 3'. F= forward, M= deleted sequence, R= reverse, WT= normal sequence.

Supp. Materials and Methods

Ethical approval was given for WGS by the board of the Medical Ethical Committee Rotterdam (MEC-2012-140) and for targeted *TCF12* sequencing by the Oxfordshire Research Ethics Committee B (reference C02.143) and London Riverside Research Ethics Committee (reference 09/H0706/20). Informed consent was received from the study participants.

Within the framework of a broader study into the genetic causes of craniosynostosis, WGS was applied on the DNA of 18 Dutch index-cases with coronal synostosis and negative testing for *FGFR2*, *FGFR3* and *TWIST1,* and their family-members (43 samples in total), by Complete Genomics, a BGI company (Mountain View, CA, USA), as described by Drmanac et al. (Drmanac, et al., 2010). Data were analyzed using cga tools version 1.6.0.43. An autosomal dominant disease model was tested. The analysis was restricted to novel non-synonymous variants, variants disrupting a splice site (±2 bp), and insertions or deletions in the coding sequence (±50 bp). Because there were no obvious mutations in known craniosynostosis genes (list of variants available on request), a structural variant analysis was performed using a custom-made Python script. Structural variant calling was performed using uniquely mapped discordant read pairs as described by Gilissen et al. (Gilissen, et al., 2014), and filtering was based on scrutinizing regions with a known association with craniosynostosis. In three families, large intragenic exon deletions were identified in *TCF12*. Variants of family 1 and 2 were annotated using NCBI build 36.3/hg18 and dbSNP build 130, variants of family 3 were annotated using GRCh37/hg19. Annotations of the deletions of family 1 and 2 were lifted over to hg19 by using Human BLAT search on the UCSC website (Kent Informatics, Inc., Santa Cruz, CA, USA).

The deletions identified by WGS were confirmed by deletion specific PCR. Primers used for the deletion PCR are given in Supp. Table S1. For each specific deletion PCR, one forward

and one reverse primer were designed outside the deletion. The third primer was designed in the deleted region in such a way that the PCR product resulting from the mutant allele was smaller than the PCR product produced from the wild-type allele.

In parallel to this, targeted sequencing of DNA samples of 160 British unrelated subjects with craniosynostosis, including the coronal suture, and previously negative testing of the *FGFR2*, *FGFR3*, *TWIST1* and *TCF12* genes, was carried out by capture of the *TCF12* genomic region (chr15: 57,029,979-57,670,037) using the SeqCap EZ Choice Library system (Roche-Nimblegen, Inc., Madison, WI, USA). Samples were multiplexed and two pools of 80 samples were each run on a single lane of the HiSeq2500 platform (Illumina, Inc., San Diego, CA, USA) run in rapid mode. The resulting data were aligned using Novoalign (http://www.novocraft.com/products/novoalign/) to the human reference genome GRCh37/hg19, and indels were detected by Pindel v0.2.4 (Ye, et al., 2009).

The duplication was confirmed on genomic DNA by breakpoint spanning PCR (primers shown in Table 1). The effect of the duplication and the deletion in family 3 were studied by cDNA analysis. RNA was extracted from a lymphoblastoid cell line obtained from individual III.1 (family 4) using Trizol reagent (Invitrogen, Carlsbad, CA, USA) and the RNeasy kit (Qiagen Ltd., Crawley, UK). cDNA was synthesized using the RevertAid First Strand cDNA kit (Thermo Scientific Inc., Waltham, MA, USA), with random hexamer primers according to the manufacturer's instructions.

All amplification reactions were performed according to standard procedures. Products were electrophoresed on agarose gels in TBE buffer. To confirm the exact position of the breakpoints, the PCR products were sequenced. PCR products were purified with ExoSAP-IT (USB, Affymetrix, Cleveland, OH, USA). Dideoxy-sequencing of both strands was performed

using Big Dye terminator version 3.1 (Applied Biosystems, Foster City, CA, USA) as recommended by the manufacturer. Dye terminators were removed using SephadexG50 (GE Healthcare, Pittsburgh, PA, USA) and loaded on an ABI 3130XL Genetic Analyzer or ABI 3730 (Applied Biosystems).

The pathogenic mutations were described according to HGVS nomenclature (den Dunnen and Antonarakis, 2000), using reference NM_207037.1 consisting of 21 exons, protein coding from exon 2 through 20, on GRCh37 and submitted to the Leiden Open Variation Database (http://www.lovd.nl/TCF12).

Subject descriptions

Family 1. The index patient was a boy, born by caesarean section due to breech position at 37+6 weeks of gestation, weighing 3075 grams (Supp. Figure S1A, III.1). He had non-consanguineous parents. Directly after birth, a progressive skull malformation was observed. Physical examination at the age of 4.5 months showed a frontal plagiocephalic head shape and a closed fontanel. The skull circumference was 42.5 cm (-0.41 SD). Apart from bilateral camptodactyly of the fifth fingers, no hand or foot anomalies were present. Skull radiographs and 3-dimensional computed tomography (3D-CT) showed synostosis of the right coronal suture. A fronto-supraorbital remodeling was performed at the age of 10 months. Furthermore, a tenotomy was performed at the age of 4 years because of divergent strabismus and an adenoidectomy was performed because of recurrent airway infections. In addition to this, he suffered from febrile seizures. He had mild learning problems (Verbal IQ 82, Performance IQ 64, Global IQ 74). Karyotyping was normal and *FGFR2*, *FGFR3*, *TWIST1* and *TCF12* were tested negative by dideoxy-sequencing. The family history was positive; his mother (II.2) was born with a left-sided

plagiocephaly, requiring a surgical correction early in life. The DNA of subjects II.1, II.2 and III.1 was sequenced.

Family 2. The index patient was a girl born as the third child of non-consanguineous unaffected parents at 42+4 weeks of gestation, weighing 3620 grams (Supp. Figure S1B, III.1). Physical examination at the age of 5 months showed a frontal brachycephalic head shape. The skull circumference was 41 cm (-0.87 SD). Her hands and feet were normal. Skull radiograph and 3D-CT showed bicoronal synostosis. At the age of 9 months, a supraorbital advancement was performed. Later, the girl was seen by an ophthalmologist because of myopia. Furthermore, she was seen by an orthodontist because of a divergent growth pattern and crowding of teeth. She had normal development. The family history was positive; a sister of the father (II.1) had an un-operated brachycephalic head shape. The father of the patient was clinically unaffected. Learning disabilities were not mentioned in the family. In the index patient, no mutations were found in *FGFR2*, *FGFR3*, *TWIST1* and *TCF12* by dideoxy-sequencing. The DNA of subjects II.1, II.2, II.3 and III.1 was sequenced.

Family 3. The index patient was a boy born as a second child at 39+4 weeks of gestation, weighing 3365 grams (Supp. Figure S1C, III.2). Physical examination at the age of 6 months showed an asymmetrical skull shape, with frontal bossing (predominantly on the left side), right-sided supra-orbital retrusion and an asymmetrical occiput. The skull circumference was 45 cm (+0.62 SD). Hands and feet were normal. Skull radiographs and 3D-CT showed premature fusion of all calvarial sutures, except for the metopic suture. Clinically, the patient was suspected to have Crouzon syndrome. A fronto-biparietal remodeling was performed at the age of 8 months.

At the age of two years, the patient had a skull circumference of 51 cm (+1.07 SD), a bony defect occipitally, and mild fingerprinting on the skull radiograph. No other symptoms of increased intracranial pressure were noticed. However, fundoscopy showed papilledema of 1.5-2 dpt. Thus, a parieto-occipital decompression was performed. During the subsequent four-year follow-up, papilledema was absent. The family history was negative. No mutations were found in *FGFR2*, *FGFR3* or *TWIST1*. The DNA of subjects II.1, II.2 and III.2 was sequenced.

Family 4. The index patient was a boy born at 38 weeks' gestation following an uneventful pregnancy (Supp. Figure S1D, III.1). At birth he was noted to have hypertelorism and brachycephaly. The head circumference was 43 cm at the age of 8 months (-1.9 SD). At the age of 10.5 months a CT head scan showed bicoronal synostosis with a very shallow anterior cranial fossa and small facial skeleton, and a fronto-orbital advancement and remodeling procedure was performed. Physical examination at the age of 6 years revealed relatively small ears with prominent helical crura and marked bilateral 5th finger clinodactyly. He attended a normal school without additional help up to the age of 15 years. He was receiving treatment for Class II.1 dental malocclusion on a mild Class II skeletal base. His mother had a normal craniofacial examination but was noted to have generalized brachydactyly. A maternal half-brother and his daughter were reported to have flattened foreheads.

Supp. References

den Dunnen JT, Antonarakis SE. 2000. Mutation nomenclature extensions and suggestions to describe complex mutations: a discussion. Hum Mutat 15(1):7-12.

Drmanac R, Sparks AB, Callow MJ, Halpern AL, Burns NL, Kermani BG, Carnevali P, Nazarenko I, Nilsen GB, Yeung G, Dahl F, Fernandez A et al. 2010. Human genome sequencing using unchained base reads on self-assembling DNA nanoarrays. Science 327(5961):78-81.

Gilissen C, Hehir-Kwa JY, Thung DT, van de Vorst M, van Bon BW, Willemsen MH, Kwint M, Janssen IM, Hoischen A, Schenck A, Leach R, Klein R et al. 2014. Genome sequencing identifies major causes of severe intellectual disability. Nature 511(7509):344-7.

Ye K, Schulz MH, Long Q, Apweiler R, Ning Z. 2009. Pindel: a pattern growth approach to detect break points of large deletions and medium sized insertions from paired-end short reads. Bioinformatics 25(21):2865-71.
